# Increased FluoroDeoxyGlucose (FDG) Avidity Following COVID-19 Vaccination

**DOI:** 10.3390/arm90050047

**Published:** 2022-08-24

**Authors:** Mo’ath Nassar, Ayman Soubani

**Affiliations:** Division of Pulmonary, Critical Care and Sleep Medicine, Wayne State University School of Medicine, Detroit, MI 48201, USA

**Keywords:** COVID-19 vaccination, complications, axillary lymphadenopathy

A 65-year-old woman presented to the Pulmonary Clinic for evaluation after Positron Emission Tomography/Computed Tomography (PET/CT), which was obtained for assessment of a 12 mm right middle lobe solitary pulmonary nodule. She denied shortness of breath, fever, arm swelling, or breast masses. The PET/CT showed intensely FluoroDeoxyGlucose (FDG) avid right axillary lymph nodes (The largest of which measured 30 mm) and the right deltoid muscle (Figure 1 and Figure 2). There was no FDG uptake in the right lung or breast. The enlarged right axillary lymph nodes were not visualized on Computed Tomography imaging obtained two weeks prior to PET/CT. A mammogram performed ten days prior also showed benign findings. The patient reported receiving the first dose of a COVID-19 vaccine one day prior to PET/CT. Right axillary ultrasonography was performed four weeks later and showed complete resolution of lymphadenopathy. The PET/CT findings were thought to be secondary to the COVID-19 vaccination [1,2].

## Figures and Tables

**Figure 1:**
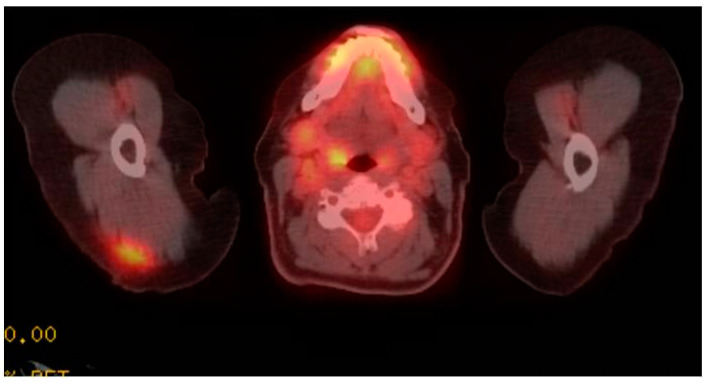
PET/CT imaging shows increased FDG avidity in right deltoid muscle.

**Figure 2:**
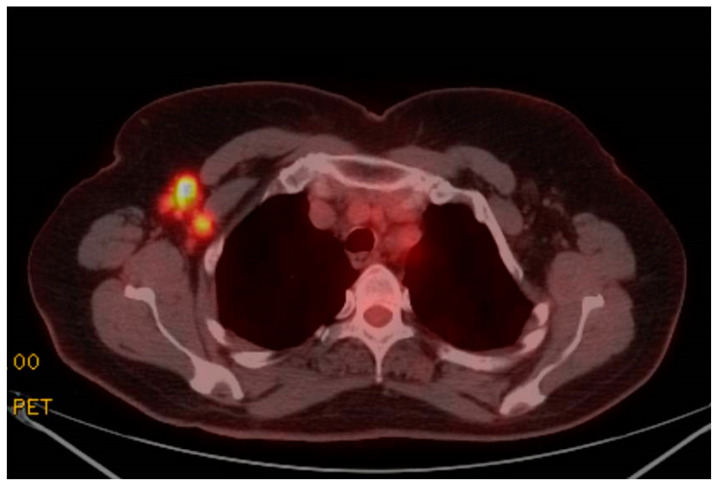
PET/CT imaging shows increased FDG avidity in right axillary lymph nodes.
